# Laboratory Determined Sugar Content and Composition of Commercial Infant Formulas, Baby Foods and Common Grocery Items Targeted to Children

**DOI:** 10.3390/nu7075254

**Published:** 2015-07-16

**Authors:** Ryan W. Walker, Michael I. Goran

**Affiliations:** 1 Department of Preventive Medicine, Icahn School of Medicine at Mount Sinai, New York, NY 10025, USA; E-Mail: ryan.walker@mssm.edu; 2 Department of Preventive Medicine, University of Southern California, Keck School of Medicine, Los Angeles, CA 90089, USA

**Keywords:** sugar, formula, high fructose corn syrup, HFCS, obesity, fructose, breastfeeding

## Abstract

Excess added sugar consumption is tied to poor health outcomes in children. The sugar content of beverages and foods children are exposed to is mostly unknown, yet this information is imperative for understanding potential risks from overconsumption of sugars in early life. We determined actual sugar content by conducting a blinded laboratory analysis in infant formulas, breakfast cereals, packaged baked goods and yogurts. One hundred samples were sent to an independent laboratory for analysis via gas chromatography. Sugar content and composition was determined and total sugar was compared against nutrition labels. Of the 100 samples analyzed, 74% contained ≥20% of total calories per serving from added sugars. Nutrient label data underestimated or overestimated actual sugars and ~25% of all samples had actual total sugar values that were either <10% or >10% of labeled total sugar. Many products that are frequently marketed to and consumed by infants and young children contain sugars in amounts that differ from nutrition labels and often in excess of recommended daily levels. These findings provide further support for adding more comprehensive sugar labeling to food and beverage products, specifically those marketed to, or commonly consumed by, children.

## 1. Introduction

Nearly 15% of the United States population consumes upwards of 25% of their total daily calories from added sugars [1]. Excess consumption of sugar sweetened beverages, a major source of added sugars, is tied to poor health outcomes in children [2], but intake is commonly measured via diet recalls or food frequency questionnaires that assume sugar values based on databases created from product nutrition labels [3,4,5]. There is very limited data from laboratory-determined measures of sugar content and little is known about the actual sugar content of common processed foods that children may be exposed to very early in life.

Nutrition and dietary habits during infancy and into early childhood play a role in shaping eating habits and health later in adolescence and into adulthood [6,7]. The effect of breastfeeding *versus* formula feeding on child health outcomes has been studied extensively and is it well established that human milk and infant formulas differ in terms of both nutrition and biological constituents [8,9]. Some formulas contain added sugars that are not present in breastmilk and the actual sugar content, in terms of both type and proportion, of infant formula is not widely known.

As children are introduced to solid foods at weaning, they may be exposed to additional processed food products that contain added sugars [10]. Like some formulas, solid foods may contain sucrose and other sugars that are not present in breastmilk. Commercial baby foods and other common grocery items that children are often exposed to in infancy can be a source of added sugars, which contribute to total daily sugar exposure. Nutrition labels for some commercial products may not always reflect the true, or most accurate, sugar content information [11,12]. Given the recent scientific, federal and consumer interest in the sugar content of foods and beverages, specifically added sugars, it is important to establish actual sugar content and composition for infant formulas and other food products children may be exposed to in early life.

Therefore, we sought to determine actual sugar content and composition, by conducting a blinded gas chromatography analysis, in 20 commonly used infant formulas, 20 baby foods and 60 other common grocery items. Several products frequently marketed towards children, based upon advertising and product packaging [13], were included in the analyses. The additional grocery categories were breakfast cereals, pre-packaged baked goods and yogurts.

## 2. Materials and Methods

One hundred food and beverage samples were selected from infant formulas and other standard grocery categories: Baby food, yogurt, breakfast cereal, and packaged baked goods. Online shopping databases for three of the Nation’s largest grocery retailers—Walmart, SuperValu, and Safeway—were accessed in order to select category-specific samples. To control for location and inventory, online store inventories were accessed for selected Los Angeles County outlets of each retailer in a defined zip code region (90033). Twenty products were selected for each of the grocery categories by choosing every tenth product in the retailers’ databases until 10 products made with high fructose corn syrup (HFCS) and 10 products made without HFCS, according to package ingredient labels, were selected. In categories where HFCS was not a commonly occurring ingredient, 10 sucrose-containing products and 10 non-sucrose-containing products, according to package ingredients labels, were selected using the same method whenever possible. An aliquot was taken directly from each product, in its original packaging, and transferred to sterile, sealed containers. Sample weights were determined and recorded. Sample weights ranged from 15 to 40 g. Samples were packaged and shipped overnight on dry ice to Covance Laboratories (Madison, WI, USA) for subsequent blinded analysis via gas chromatography (Agilent 6890N), against internal standards, according to previously published methods [14,15,16].

The sugar profile analysis conducted at Covance was applicable to the determination of fructose, galactose, glucose, sucrose, lactose, and maltose in as little as 10 g of food products, syrups, and beverages. Once received, samples were prepared in accordance with Covance procedures and sugars were extracted from the homogenized sample with water. Aliquots were dried under inert gas and reconstituted with a hydroxylamine hydrochloride solution in pyridine containing phenyl-β-d-glucoside as the internal standard. The resulting oximes were converted to silyl derivatives with hexamethyldisilazane and trifluoracetic acid treatment and analyzed by gas chromatography [15,16] using a flame ionization detector. This methodology does not acid hydrolyze or purposefully degrade sugars during analysis, thereby mitigating the risk of more complex sugars degrading during analysis (the sugars are in solution and the extracting solvents inhibit enzymatic activity). All GC sample analyses were conducted with an internal standard (Phenyl-Beta-d-Glucopyranoside). An additional 10% of each sample analytical run was tested in duplicate and validated against two internally-validated control standards. The limit of quantitation for most matrices is 0.1%. A cereal product was included in daily batch analyses as a routine control material. A control chart was created for these results and values were used as a quality control parameter for the acceptance of GC assay data. RSDs (relative standard deviation) were assembled from many data points and provide a reliable representation of the variance for the cereal product matrix across days, instruments and analysts. The cereal product RSDs for fructose, glucose, sucrose, and maltose were 4.9, 7.4, 3.2, and 6.4, respectively. Results underwent further quality control comparison with internal validated controls, linearity expectations and historical data.

### Analysis

Sugar data was provided in a value of grams per 100 g of sample, as per industry standard [17]. Data for individual sugars were converted for reporting into the following formats; percent of total sugar and concentration of each sugar in grams per serving (g/s). Actual total sugar was calculated. The percent of total calories per serving (based upon nutrition label values, where available) from sugar was calculated. Formulas used to obtain these values are presented in Table S1. Products with “added sugar” listed one or more of the following sugars in the product nutrition label ingredients; HFCS, sucrose, fructose, glucose, lactose, glucose, galactose, maltose or corn syrup. The percent of total sugar from “added” *vs.* “intrinsic” sources was determined (Table S2). The percent of total sugar from added sugar is equal to the percentage sum of all measured sugars that were listed in the product ingredients. The percent of total sugar from intrinsic sugar is equal to all other measured sugars (not listed in product ingredients). In products listing “sugar” as an ingredient, sucrose was included as an added sugar. Maltose was included as an added sugar in items listing corn syrup as an ingredient. Glucose and fructose were included as added sugars in products listing HFCS on the label. In fruit-based products containing HFCS it was not possible to determine proportions of fructose and glucose from added *vs.* intrinsic sources.

## 3. Results

### 3.1. Sugar Analyses Using Gas Chromatography in 100 Foods and Beverages

#### 3.1.1. Infant Formula

**Figure 1:**
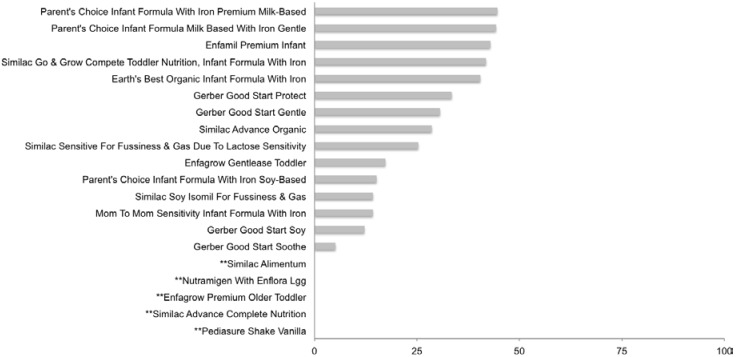
Percent of total calories from sugar: Formula. ** Unable to calculate in premixed, ready to use samples. Values were derived from the following equation: % Total Calories from sugar = [(Total Sugar_Actual_ per serving × 4)/Total Calories per serving] × 100.

Mean percent of total calories per serving from sugars was 27.4% ± 13.6%. Five formulas had >40% of total calories from sugars (Figure 1). Sugar content was predominantly distributed amongst lactose, sucrose, maltose or glucose (77.5 ± 34, 68.6 ± 28.5, 19.1 ± 24.7 and 6.6 ± 10.1%, respectively), in products that contained said sugars, with little to no free fructose or galactose detected in most products (Table 1). Formulas highest in sucrose were alternatives to lactose-containing milk-based formulas. All but two formulas listed sugars on the ingredients label, thus qualifying them as products with added sugar. Among products with added sugar mean percent of total sugar from added sugar sources was 69.9 ± 37.4% (Table S2). Grams of total sugars per serving ranged from 1.28 to 11.16 g, with five products containing >10 g of sugar per serving (Table S3).

#### 3.1.2. Baby Food

**Figure 2:**
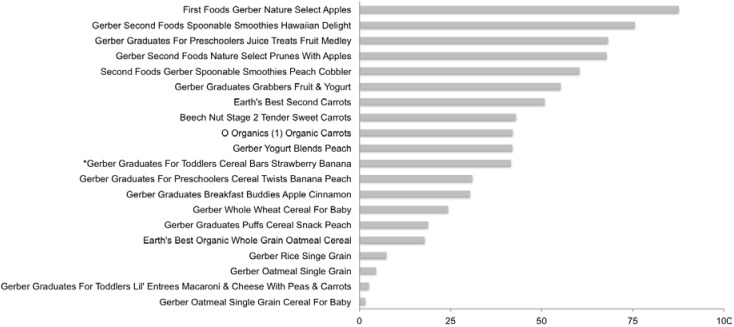
Percent of total calories from sugar: Baby Food. Values were derived from the following equation: % Total Calories from sugar = [(Total Sugar_Actual_ per serving × 4)/Total Calories per serving] × 100.

Mean percent of total calories per serving from sugars was 38.6 ± 25.5% and seven of the 20 baby food products contained >50% of their total calories from sugar (Figure 2) with First Foods Gerber Select Apples having 87.6% of its total calories from sugar. Several of the fruit-based products had >40% of total sugars from free fructose (Table 1), with First Foods Gerber Select Apples having 68.5% of total sugars from free fructose. The remainder of baby foods tested had free fructose levels below 55%. Seven baby foods listed sugars on the ingredients label and the mean percent of total sugar from added sugar sources was 53.6 ± 24.1% (Table S2). Gerber Spoonable Smoothies Hawaiian Delight contained 20.7 g of total sugars per serving and six other products has >10 g/s of sugar (Table S3). Only one product listed HFCS as a sweetener.

**Table 1:** Percentage of Total Sugar.

Formula	% Fructose	% Glucose	% Sucrose	% Maltose	% Galactose	% Lactose
Pediasure Shake Vanilla	0.00	1.37	98.63	0.00	0.00	0.00
Similac Alimentum	0.00	0.00	100.00	0.00	0.00	0.00
Enfagrow Premium Older Toddler	0.00	1.85	14.81	0.00	0.00	83.33
Similac Advance Complete Nutrition	0.00	1.59	0.00	0.00	0.00	98.41
Similac Go & Grow Compete Toddler Nutrition, Infant Formula With Iron	0.00	2.08	0.00	0.00	0.21	97.71
Earth’s Best Organic Infant Formula With Iron	0.00	0.65	0.00	3.44	0.00	95.91
Nutramigen With Enflora Lgg	0.00	42.22	0.00	57.78	0.00	0.00
Enfagrow Gentlease Toddler	0.00	10.05	0.00	11.06	0.50	78.39
Gerber Good Start Soothe	0.00	20.34	0.00	79.66	0.00	0.00
Parent’s Choice Infant Formula with Iron Soy-Based	0.00	1.72	0.00	4.60	2.87	90.80
Parent’s Choice Infant Formula with Iron Premium Milk-Based	0.00	1.75	0.00	0.00	0.19	98.05
Parent’s Choice Infant Formula Milk Based with Iron Gentle	0.00	1.77	0.00	0.00	0.20	98.04
Gerber Good Start Protect	0.00	1.04	0.00	1.30	1.04	96.62
Gerber Good Start Gentle	0.00	3.69	0.00	1.70	1.70	92.90
Enfamil Premium Infant	0.00	1.22	0.00	0.00	0.41	98.38
Mom To Mom Sensitivity Infant Formula With Iron	0.56	6.67	63.89	24.44	0.00	4.44
Similac Advance Organic	0.61	1.52	44.68	3.34	0.00	49.85
Similaac Sensitive For Fussiness & Gas Due To Lactose Sensitivity	0.69	9.28	79.73	8.25	0.00	2.06
Gerber Good Start Soy	0.71	3.57	82.14	13.57	0.00	0.00
Similac Soy Isomil For Fussiness & Gas	1.22	14.02	64.63	20.12	0.00	0.00
**Yogurt**	**% Fructose**	**% Glucose**	**% Sucrose**	**% Maltose**	**% Galactose**	**% Lactose**
Dannon Oikos Blueberry	46.09	9.38	24.22	0.00	4.69	15.63
Great Value N.F. Strawberry Banana	42.37	3.39	0.00	0.00	11.86	42.37
Dannon Oikos Greek N.F. Vanilla	40.17	2.56	36.75	0.00	5.13	15.38
Activia Light Strawberry	30.99	4.23	2.82	0.00	5.63	56.34
Activia Vanilla	27.97	2.80	44.76	0.00	2.80	21.68
Lucerne L.F. Tubes Blueberry Bubblegum	26.28	1.28	44.87	0.00	2.56	25.00
Lucerne L.F. Tubes Strawberry	25.81	0.65	45.81	0.00	2.58	25.16
* Yoplait Whips! Chocolate	20.71	9.09	48.48	0.00	2.53	19.19
Lucerne L.F. Yogurt Peach	11.03	9.56	57.35	0.00	3.68	18.38
Chobani Raspberry	10.26	8.55	58.97	0.00	4.27	17.95
Yoplait Light Blackberry	3.70	3.70	37.04	0.00	7.41	48.15
Yoplait Original Strawberry	2.50	2.50	72.50	0.00	3.13	19.38
Dannon Danimal strawberry-Banana Flavored	2.19	2.19	76.64	0.00	3.65	15.33
Yoplait Gogurt Perry Berry	1.17	1.17	79.53	0.00	2.92	15.20
Yoplait Trix Strawberry Banana Bash	0.74	1.47	72.79	0.00	2.94	22.06
Yoplait Gogurt Summer Punch	0.58	1.16	79.77	0.00	2.89	15.61
Yoplait Light Key Lime Pie	0.00	0.00	39.29	0.00	7.14	53.57
Yoplait Light Banana Cream Pie	0.00	1.54	40.00	0.00	7.69	50.77
Yoplait Light Very Vanilla	0.00	1.59	39.68	0.00	7.94	50.79
**Baked Goods**	**% Fructose**	**% Glucose**	**% Sucrose**	**% Maltose**	**% Galactose**	**% Lactose**
* Keebler 100 Calorie Right Bites	46.88	17.81	27.81	5.94	0.00	1.56
Kellogg’s Special K Pastry	33.57	12.94	45.80	6.64	0.35	0.70
* Nabisco Fig Newtons	29.01	35.62	26.72	8.14	0.51	0.00
Fiber One 90 Calorie Brownies	22.61	1.91	75.16	0.32	0.00	0.00
* Kellogg’s Pop-Tarts Strawberry	14.38	52.61	22.88	10.13	0.00	0.00
* Hostess Twinkies	11.23	33.68	42.82	9.92	0.00	2.35
* Little Debbie Muffins Blueberry	8.38	17.30	71.62	1.08	0.00	1.62
* Safeway Graham Crackers	8.24	7.84	82.75	0.00	1.18	0.00
Entenmann’s Little Bites	7.09	2.75	84.67	3.20	0.00	2.29
* Nabisco Nilla Wafers	4.48	5.07	86.87	0.00	0.00	3.58
* Bimbo Mini Muffins	3.65	4.38	89.78	0.36	0.00	1.82
* Hostess Sno Balls	3.60	18.65	65.62	11.46	0.00	0.67
* Little Debbie Cosmic Brownies	2.65	38.73	46.95	11.67	0.00	0.00
* Mini Oreo Bite Size	2.61	10.97	86.42	0.00	0.00	0.00
* Mini Chips Ahoy Bite Size	1.93	4.18	93.89	0.00	0.00	0.00
Hostess Ho Hos	1.79	4.56	88.10	2.78	0.00	2.78
Entenmann’s Buttermilk Donuts	0.99	2.47	94.32	0.74	0.00	1.48
* Hostess Donettes Crunch	0.92	3.99	89.88	3.99	0.00	1.23
Hostess Donettes	0.74	41.91	52.94	2.57	0.00	1.84
**Cereals**	**% Fructose**	**% Glucose**	**% Sucrose**	**% Maltose**	**% Galactose**	**% Lactose**
Cinnamon Toast Crunch	31.39	5.50	63.11	0.00	0.00	0.00
Corn Flakes	15.48	14.29	59.52	10.71	0.00	0.00
* Essential Everyday Crispy Rice	8.24	9.41	78.82	3.53	0.00	0.00
Honey Nut Cheerios	6.07	6.07	87.86	0.00	0.00	0.00
Kellogg’s Special K	4.13	3.31	86.78	4.96	0.00	0.83
Kix	3.96	2.97	93.07	0.00	0.00	0.00
Golden Grahams	3.70	3.99	91.74	0.00	0.57	0.00
Honey Bunches Of Oats	3.68	5.26	81.58	8.42	0.00	1.05
Kashi Go Lean Crunch	3.64	6.36	76.36	13.64	0.00	0.00
Kellogg’s Frosted Flakes	3.27	3.78	91.94	1.01	0.00	0.00
Safeway Crispy Rice	2.86	3.81	89.52	3.81	0.00	0.00
Rice Chex	2.70	2.70	94.59	0.00	0.00	0.00
Cocoa Puffs	2.54	6.09	87.56	3.81	0.00	0.00
Cheerios	2.50	0.00	97.50	0.00	0.00	0.00
Trix	2.48	10.56	81.37	5.59	0.00	0.00
Lucky Charms	2.06	13.53	79.59	4.82	0.00	0.00
Frosted Cheerios	1.16	4.64	91.88	2.32	0.00	0.00
Frosted Mini Wheats Little Bites	1.00	1.50	95.00	2.50	0.00	0.00
Kellogg’s Froot Loops	0.98	1.22	97.80	0.00	0.00	0.00
Life	0.94	1.41	93.90	3.76	0.00	0.00
**Baby Foods**	**% Fructose**	**% Glucose**	**% Sucrose**	**% Maltose**	**% Galactose**	**% Lactose**
Gerber Nature Select Apples	68.52	23.15	8.33	0.00	0.00	0.00
Gerber Prunes With Apples	54.17	42.50	3.33	0.00	0.00	0.00
Gerber Smoothies Peach Cobbler	46.72	40.16	13.11	0.00	0.00	0.00
Gerber Breakfast Apple Cinnamon	42.17	31.33	26.51	0.00	0.00	0.00
Gerber Smoothies Hawaiian Delight	39.05	35.24	18.10	0.00	0.48	7.14
Gerber Grabbers Fruit & Yogurt	34.78	18.26	34.78	0.00	2.61	9.57
Earth’s Best Second Carrots	24.44	24.44	51.11	0.00	0.00	0.00
* Gerber Cereal Bars Strawberry Banana	22.98	32.11	42.04	2.35	0.00	0.52
O Organics (1) Organic Carrots	13.51	16.22	70.27	0.00	0.00	0.00
Beech Nut Tender Sweet Carrots	13.16	13.16	73.68	0.00	0.00	0.00
Gerber Juice Treats Fruit Medley	12.64	20.69	59.93	6.73	0.00	0.00
Gerber Cereal Twists Banana Peach	10.65	19.35	61.94	5.48	0.00	2.58
Gerber Puffs Cereal Snack Peach	10.12	3.57	86.31	0.00	0.00	0.00
Gerber Yogurt Blends Peach	4.72	3.77	53.77	0.00	6.60	31.13
Gerber Whole Wheat Cereal (Baby)	0.44	1.32	4.82	93.42	0.00	0.00
Gerber Oatmeal Single Grain	0.00	0.00	22.22	77.78	0.00	0.00
Gerber Rice Singe Grain	0.00	42.03	2.90	55.07	0.00	0.00
Earth’s Best Organic Whole Grain Oatmeal	0.00	1.25	5.63	93.13	0.00	0.00
Gerber Oatmeal Single Grain Cereal (Baby)	0.00	0.00	56.25	43.75	0.00	0.00

Values for each sugar represent percentage of total sugar in product; * products listing HFCS as an ingredient.

#### 3.1.3. Yogurt

**Figure 3:**
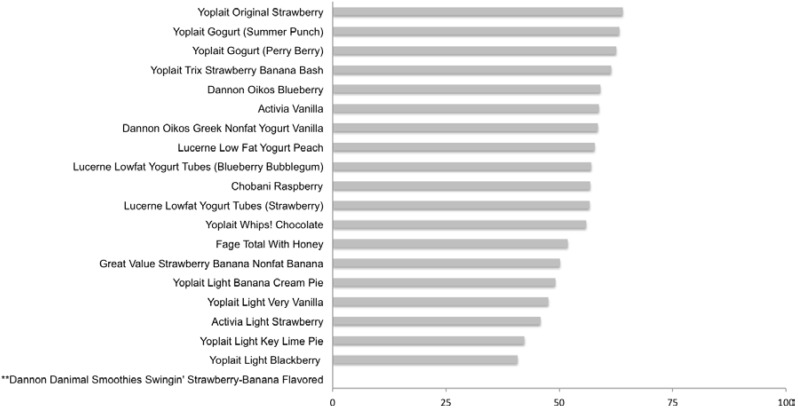
Percent of total calories from sugar: Yogurt. ** Label specifies no sugar. Values were derived from the following equation: % Total Calories from sugar = [(Total Sugar_Actual_ per serving × 4)/Total Calories per serving] × 100.

Mean percent of total calories per serving from sugars in the 20 yogurt samples tested was 51.9 ± 13.9%. Fourteen products contained >50% of their total calories from sugar (Figure 3). Many yogurt products were sweetened with multiple sugars (sucrose, fructose and/or HFCS) and percentages of total sugars are listed in Table 1. All yogurts (excepting Chobani Raaspbery) contained added sugars with mean percent of total sugar from added sugar sources being 63.3 ± 17.8% (Table S1). Yoplait Original Strawberry had a total sugars per serving of 27.2 g/s and the mean sugar content per serving for all products was 14.4 ± 5.6 g/s (Table S3). The yogurt products analyzed ranged from 0 to 46% free fructose (Table 1). Nine of the 20 products tested reported fructose, of which one also listed HFCS, as an ingredient.

#### 3.1.4. Breakfast Cereals

Mean percent of total calories per serving from sugars was 24.9 ± 13.7% and twelve products contained >20% of their total calories from sugar (Figure S1). The majority of products were mainly sweetened with sucrose (mean % sucrose was 85.9 ± 10.5%), however two products (Cinnamon Toast Crunch and Corn Flakes) that did not list HFCS as an ingredient contained free fructose in excess of 15% of total sugars (Table 1). All cereal products (excepting Kashi Go Lean Crunch) contained added sugars with mean percent of total sugar from added sugar sources being 90.5 ± 8.4% (Table 2). Most cereal products contained less than 10 g of total sugars per serving, however Kellogg’s Frosted Flakes contained 23.8 g/s of total sugar (Table S3). Only one cereal product listed HFCS as an ingredient (Essential Everyday Crispy Rice).

#### 3.1.5. Pre-Packaged Baked Goods

Mean percent of total calories per serving from sugar was 34.9 ± 7.6% with all products containing >20% of their total calories from sugars (Figure S2). Baked goods were primarily sweetened with sucrose (mean % of total sugars from sucrose was 67.1 ± 24.9% (Table 1)). All baked goods products contained added sugars with mean percent of total sugar from added sugar sources being 93.4 ± 11.6% (Table S2). Mean total sugars per serving in baked goods was relatively high at 18.9 ± 11.8 g/s and four products (Hostess Twinkies, Hostess Donettes Crunch, Hostess Ho Hos and Hostess Sno Balls) had total sugars values of ≥30 g/s (Table S3). Low-calorie optioned baked goods (Keebler Right Bites and Fiber One brownies) tended to have higher fructose than sucrose levels.

### 3.2. Comparison of Gas Chromatography Measured Total Sugar with Nutrient Label Data

Total sugars per serving was calculated for all products, using the equation listed in Table S1, for comparison with per serving values listed on the individual product nutrient labels. This comparison could not be made in a subset of products due to no sugar per serving values being present on the label (formulas), specific gravity not being measured (one yogurt product) or the label specifying zero sugar (one baby food product). The percent difference from labeled sugar varied widely within and between grocery product groups (Table 2). As mentioned, formulas did not provide sugar per serving values on nutrition labels preventing the comparison in this group. Baby foods ranged from 88% less to 82% more total sugar than listed and similar observations were made for yogurts (24% less to 11% more), breakfast cereals (21% less to 18% more) and packaged baked goods (15% less to 28% more).

**Table 2:** Comparison of per serving sugar: Laboratory measured *vs.* nutrition label value.

Infant Formula	% Difference From Label
* NA	* NA
Baby Foods	% Difference From Label
Gerber Whole Wheat Cereal For Baby	82.40
O Organics (1) Organic Carrots	31.35
Gerber Graduates Puffs Cereal Snack Peach	17.60
Gerber Rice Singe Grain	10.40
First Foods Gerber Nature Select Apples	9.54
Gerber Second Foods Spoonable Smoothies Hawaiian Delight	3.95
Earth’s Best Second Carrots	1.70
Gerber Graduates For Preschoolers Juice Treats Fruit Medley	0.31
Gerber Graduates Breakfast Buddies Apple Cinnamon	−3.42
Gerber Yogurt Blends Peach	−4.60
Second Foods Gerber Spoonable Smoothies Peach Cobbler	−7.09
Gerber Graduates Grabbers Fruit & Yogurt	−8.00
Gerber Second Foods Nature Select Prunes With Apples	−8.62
Gerber Graduates For Toddlers Cereal Bars Strawberry Banana	−9.04
Gerber Graduates For Preschoolers Cereal Twists Banana Peach	−11.43
Beech Nut Stage 2 Tender Sweet Carrots	−14.12
Gerber Oatmeal Single Grain	−66.25
Gerber Graduates For Toddlers Lil’ Entrees Macaroni & Cheese With Peas & Carrots	−70.00
Gerber Oatmeal Single Grain Cereal For Baby	−88.00
** Earth’s Best Organic Whole Grain Oatmeal Cereal	-
**Yogurt**	**% Difference From Label**
Yoplait Gogurt (Summer Punch)	10.72
Yoplait Light Banana Cream Pie	10.50
Yoplait Trix Strawberry Banana Bash	9.77
Yoplait Gogurt (Perry Berry)	9.44
Yoplait Original Strawberry	8.80
Yoplait Light Very Vanilla	7.10
Chobani Raspberry	4.68
Yoplait Whips! Chocolate	1.70
Dannon Oikos Blueberry	1.05
Activia Light Strawberry	0.29
Lucerne Lowfat Yogurt Tubes (Blueberry Bubblegum)	−0.16
Lucerne Lowfat Yogurt Tubes (Strawberry)	−0.80
Dannon Oikos Greek Nonfat Yogurt Vanilla	−2.50
Lucerne Low Fat Yogurt Peach	−3.67
Yoplait Light Key Lime Pie	−4.80
Activia Vanilla	−4.95
Yoplait Light Blackberry	−8.20
Great Value Strawberry Banana Nonfat Banana	−8.82
Fage Total With Honey	−23.97
*** Dannon Danimal Smoothies Swingin’ Strawberry-Banana Flavored	-
**Breakfast Cereal**	**% Difference From Label**
Lucky Charms	17.72
Life	13.60
Kellogg’s Frosted Flakes	13.43
Cheerios	12.00
Golden Grahams	8.81
Frosted Cheerios	7.33
Cocoa Puffs	6.38
Kix	1.00
Honey Nut Cheerios	0.65
Cinnamon Toast Crunch	0.42
Frosted Mini Wheats Little Bites	0.00
Rice Chex	−0.10
Trix	−0.18
Kellogg’s Froot Loops	−4.57
Honey Bunches Of Oats	−5.00
Kellogg’s Special K	−6.22
Essential Everyday Crispy Rice	−6.50
Kashi Go Lean Crunch	−10.31
Safeway Kitchens Crispy Rice	−13.38
Corn Flakes	−21.60
**Packaged Baked Goods**	**% Difference From Label**
Entenmann’s Classic Glazed Buttermilk Donuts	28.25
Hostess Donettes	19.26
Hostess Donettes Crunch	11.63
Entenmann’s Little Bites	10.59
Little Debbie Little Muffins Blueberry	8.69
Bimbo Mini Mantecadas Mini Muffins	7.89
Entenmann’s Little Bites	4.88
Mini Chips Ahoy Bite Size	3.67
Kellogg’s Special K Pastry Crisps Chocolaty Delight	2.14
Nabisco Fig Newtons	1.53
Mini Oreo Bite Size	0.97
Hostess Ho Hos	−0.37
Kellogg’s Pop-Tarts Frosted Strawberry	−0.55
Safeway Graham Crackers	−1.19
Fiber One 90 Calorie Brownies	−1.88
Little Debbie Cosmic Brownies With Chocolate Chip Candy	−2.61
100 Calorie Right Bites Keebler Mini Brownies	−4.00
Hostess Sno Balls	−4.23
Nabisco Nilla Wafers	−8.64
Hostess Twinkies	−15.74

Unable to calculate due to: * no sugar per serving value on label; ** Label specifies zero sugar; *** specific gravity not measured to determine actual sugar content per serving.

## 4. Discussion

This is the first comprehensive study to use laboratory-based chemical analyses to determine the actual content and composition of sugars in infant formulas, baby foods and a broad spectrum of popular grocery items that children are often exposed to. A principal finding of this study is that 74% of the 100 selected samples analyzed contained ≥20% of total calories per serving from sugars. Second, of the products analyzed 83% contained at least one source of added sugar and amongst these products 74% of total sugar content came from added *versus* intrinsic sugar sources. Additionally, nutrient label data often grossly under or overestimated actual sugar content, an observation most prominent in baby foods, which range from 88% less to 82% more total sugar than listed. These findings build upon prior work [11,12], which determined the sugar content of sugar sweetened beverages and juices with laboratory analyses, and further supports the concept that children may be exposed to greater than anticipated consumption of daily added sugars. Current evidence suggests that children should limit sugar intake to less than 10% of total energy [18,19]. Given our findings in the selected products analyzed, it could be difficult to achieve these recommended levels of sugar consumption. This could put children at risk of future health consequences.

Of the 20 infant formulas that were analyzed, nine contained ≥20% of total calories per serving from sugars (seven products had ≥93% of total sugar from lactose while two had ~45% and 80% of total sugar from sucrose). Although these measures are not directly comparable to the sugar content (lactose) in mature human breastmilk, which although variable generally remains close to 7% [8,20], it is important to point out the differences in sugar composition. Most formulas list sugar in their ingredients, which classifies them as products with added sugar. The added *vs.* intrinsic sugar classification is difficult to interpret in formulas, thus it is perhaps more relevant to dichotomize these products by lactose *vs.* any other added sugar. As mentioned above, in products designed to mimic the composition of breastmilk the majority of this “added” sugar comes from lactose, the naturally occurring sugar in human milk. Although free fructose detected in formulas was low, in seven products sucrose was the dominant added sugar representing nearly 86% of total added sugar. This was most notable in formulas designed for children with lactose intolerance or other gastrointestinal sensitivities. Of note, only two of the 20 infant formula products listed values for total sugars in grams per serving on their nutrition labels, making interpretation of sugar content and consumption impossible. It is not well understood how exposure to sucrose, and therefore fructose, in infancy impacts development and health progression. It is, however, well established that early life exposure to sugars other than lactose can influence taste preferences, satiety and health longitudinally [21,22] and numerous animal studies show that exposure to fructose in early-life, including maternal transmission during pregnancy and lactation, can have long-lasting obesity promoting effects in the offspring [23]. These findings become important when considering baby foods as well, given that the mean percent of total calories per serving from sugar was 38.6 ± 25.5% and seven baby food products contained >50% of their total calories from sugar. This is consistent with a recent nutrient label analysis showing that 45% of 240 packaged baby foods had sugar levels in excess of 20% of calories from sugar [24]. Given the abundance of data linking added sugar consumption to disease risk it is imperative that medical professionals and consumers understand the actual sugar content of these products so as to make informed decisions in using them to nourish children early in life.

The yogurt samples that we analyzed contained 51.9% ± 13.9% of total calories per serving from sugars. Seven of the yogurt products that were analyzed were directly marketed towards children, five of which contained >10 g of total sugars per serving (Yoplait Whips! Chocolate, Dannon Danimal Strawberry-Banana Flavored, Yoplait Gogurt Perry Berry, Yoplait Trix Strawberry Banana Bash and Yoplait Gogurt Summer Punch), which translates to over 50% of total calories per serving from sugar. The variability in fructose content and overall composition was large in yogurt products. Interestingly, several “light” or “low fat” products had more fructose than sucrose, which would be consistent with the common use of HFCS-90 as an unrestricted ingredient to sweeten low calorie foods [25] or the use of additional free fructose to improve palatability in some low-calorie food products. Lactose is a naturally occurring disaccharide (glucose and galatose) in yogurt and, as such, is expected to contribute to total sugar content. However, the predominant sugar in the yogurt products analyzed was added sucrose. Notably, in six of the seven products marketed towards children 70%–80% of total sugar comes from added sucrose and fructose whereas 15%–20% came from lactose (see Table 1). Given such high added sugar content, specifically in yogurt products for children, some of the beneficial health effects of yogurt [26,27] could be undermined by excessive sugar content.

Daily consumption of breakfast cereals is predictive of a lower body mass index [28,29,30]. However, the high sugar content (mean percent of total calories per serving from sugar was 24.9% ± 13.7%) we observe within some of these products may place children at a higher risk for poor metabolic outcomes. This is especially true given recent findings demonstrating that up to 5% of children often consume more than 10 servings per day of cereal [30]. Nineteen cereal products contained added sugar as ~91% of total sugar, predominantly in the form of sucrose. Our analyses are consistent with another recent report by the Environmental Working Group [31] that noted very high total and added sugar content in similar cereal products. Cereals may be a viable source of vitamins and fiber, but when consumed in excess, exposure to added sucrose and fructose in some products could be deleterious to metabolic health. The Children’s Food and Beverage Advertising Initiative has established maximum per-serving total sugar contents for fruit, vegetable and grain products [32]. Most cereal products exceed these recommendations that stipulate less than 10 g/s should come from sugar.

This study does have some limitations that merit discussion. The selection of the products analyzed was not based on their actual contribution to the diet of children. Unfortunately there are no data for how little or how much each of these products contribute to the diets of infants and young children. The list of products analyzed is by no means comprehensive, however it is likely representative of the average sugar profiles amongst products in each category, which is supported by another study that analyzed products in a similar category [31]. Although our data do not provide direct evidence that these products contribute to total sugar consumption in children, we feel that they strongly support our suggestion that adherence to current dietary guidelines may be difficult given the abundance of total sugar in products that children commonly consume. Our selection of samples from grocery categories was not completely random. Rather, it was based on a strategy to systematically select products with HFCS as a listed or unlisted ingredient. This could potentially bias the analysis towards higher fructose concentrations, however given the pervasive use of HFCS and sucrose as added sugars in the industry it may be a realistic representation of the actual sugar composition of products in these categories. More analyses of products will be needed to determine this. Additionally, we were unable to make added *vs.* intrinsic sugar comparisons in some products with naturally occurring sugars (fruit and dairy) and HFCS. Our use of GC methodology to analyze sugars has been previously criticized [33,34] for theoretically overestimating fructose concentrations resultant from not accurately capturing DP2+ sugars (an industry classification referring to the sum of maltose, maltotriose, and maltotetraose). Although the focus of this study is not fructose-centric, per se, it is worth noting that our prior work [11,35] has replicated sugar values (specifically fructose) across a variety of methodologies, including high performance liquid chromatography (HPLC). The White *et al.* papers posit that the HPLC method employed in their work is superior. However, the HPLC method used is an internal, industry-utilized protocol adapted from methodologies utilized by the Corn Industries Research Foundation, Corn Refiners Association and the Sweetener Technical Committee [34], and developed by the International Society of Beverage Technologists (ISBT) [36] to measure sugar composition in a beverage matrix. This is a widely used method, however it is not readily apparent that the ISBT methods have been subject to validation and critical review outside of the confines of internal food and beverage-related industrial technical publications. GC is also extensively used in industry for similar sugar analysis purposes and although we cannot directly compare against HPLC-generated values for the samples in this study, we are confident that GC accurately captures the sugars that are the focus of this work, namely sucrose, fructose, glucose, galactose, maltose and lactose. We acknowledge that the field would greatly benefit from an independent body conducting a double-blinded assessment of the sugar composition of food and beverage items in order to resolve the ongoing discrepancy in the sugar composition of popular beverages and other products that children may be exposed to, which is of paramount importance to our understanding of nutritional factors associated with the major burden of obesity and diabetes in our population.

Our study has demonstrated that total sugars, in many cases, constitute well over 20% of total calories across a variety of products (including infant formula) that children regularly consume in early life. High levels of sugar consumption, specifically fructose, have been shown to be metabolically deleterious in humans as soon as 10 weeks [37,38,39,40]. Although some products were low in measurable free fructose (not sweetened with HFCS), sucrose does contribute substantially to fructose exposure once it is enzymatically degraded. Therefore, exposure in early life to food products high in added sugars, such as sucrose or fructose, could “add up” across food categories and contribute to elevated risk for serious metabolic health outcomes [40,41]. The American Heart Association recommends less than 12 g/day added sugars for persons consuming 1600 kcal/day (representative of children 4–8 years) and the Unites States Dietary Guidelines Advisory Committee stipulates that added sugars should constitute less than 10% of total calories per day [42,43]. Thus the chronic, long-term consumption of foods and beverages high in sugar above these guidelines represents a significantly risk to child health. Of note was the large variability in actual sugar per serving content *versus* the labeled per serving sugar values. Nearly one quarter (22%) of all products included in the comparison had actual total sugar values that were either greater than 10% or less than 10% of labeled total sugar (Table 2). The massive variability detected in the actual *versus* labeled total sugar content of baby foods (±over 80% difference from label) is especially alarming. These wide ranges illustrate the enormous variability present in products, which could be due to batch differences or errors when assessing nutrient composition for reporting to the FDA [17]. Many of the baby foods indicating no added sugar in their ingredients, had high sugar content. This is possibly due to the presence of other sugar sources (brown rice syrup, evaporated cane juice, mono- and diglycerides, invert sugar and fruit or fruit juice concentrate) that are not generally highlighted as sources of added sugar. Thus, in many products listing no added sugar there are still high levels of fructose and sucrose regardless of whether the source of the sugar is added or intrinsic, which is similar to recent findings in fruit juices [11]. This collective work further supports prior concern that nutrition databases, and thus the nutrition labels that they are based upon, may not accurately reflect true sugar content. This could potentially lead to the over or underestimations of sugar intake in research and clinical contexts. More importantly, these discrepancies are apparent in some foods developed for and marketed directly towards infants and toddlers. Given these findings, informing the consumer of the sugar content of foods children are exposed to, by appropriate labeling of total sugar content in addition to actual sugar composition, could lead to judicious use of sweetened foods [44] and assist in preventing future adverse health outcomes resultant from the overconsumption of sugar early in life.

## 5. Conclusions

In conclusion, this study provides new information on the sugar composition and overall content of a multitude of commonly consumed food products. Many products that are frequently marketed to and consumed by infants and young children contain sugars that are far in excess of what is considered nutritionally beneficial and/or different from that stated on nutrition label. Given the importance of early infant nutrition in developmental programming [6,23] and exposure to excess sugar in infancy being associated with adverse metabolic consequences in adolescence and adulthood, it is essential to limit added sugar consumption in infancy and for consumers to be aware of the sugar content in products for children. These findings provide further support for adding more comprehensive sugar labeling to food and beverage products, specifically those marketed to, or commonly consumed by, children.

## Supplementary Files

Supplementary File 1
