# Supplementary material for: Laboratory Determined Sugar Content and Composition of Commercial Infant Formulas, Baby Foods and Common Grocery Items Targeted to Children
Source: Nutrients. 2015 Jul 16;7(7):5850–67. doi: 10.3390/nu7075254 (PMC4517031; doi:10.3390/nu7075254)
Supplement: Supplementary File 1 [file nutrients-07-05254-s001.docx]

**Supplementary Information**


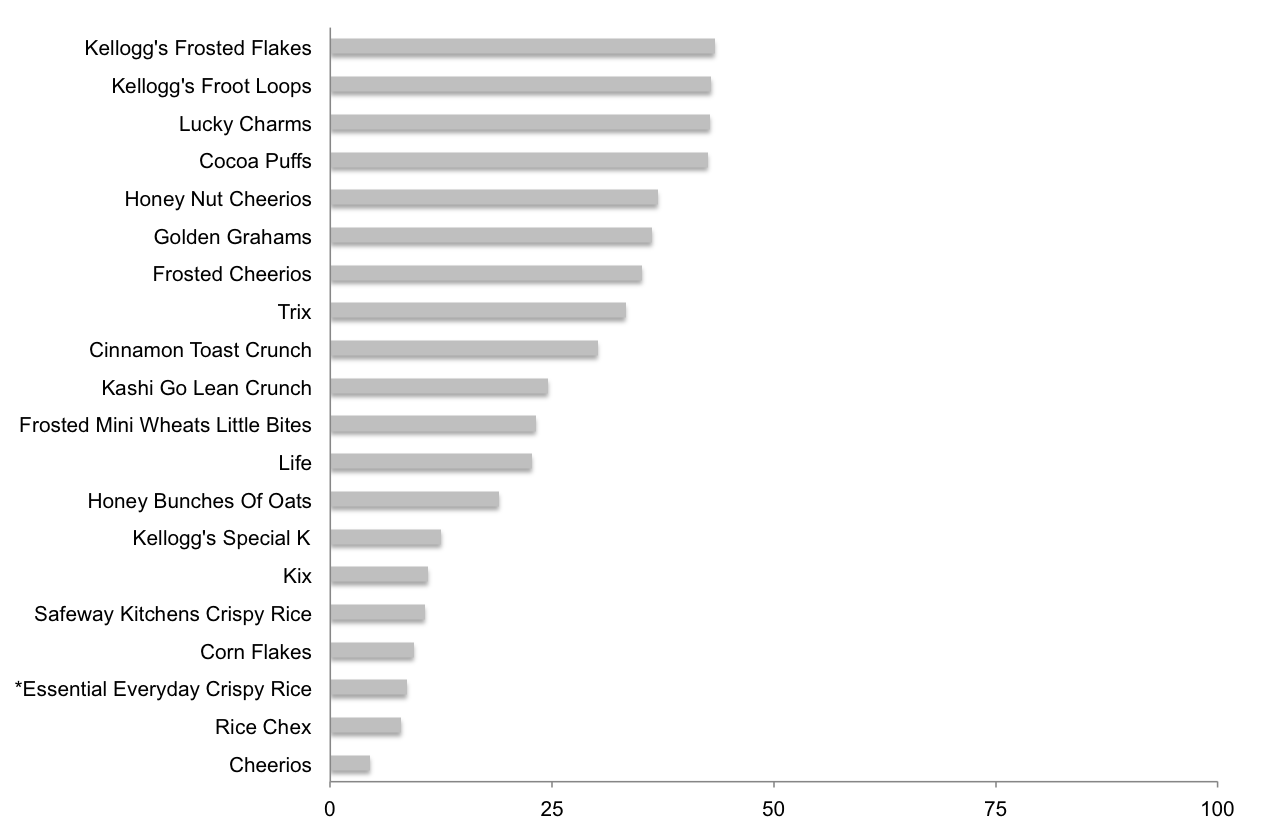


**Figure S1.** Percent of total calories from sugar: Breakfast Cereal. ***** HFCS listed as ingredient.


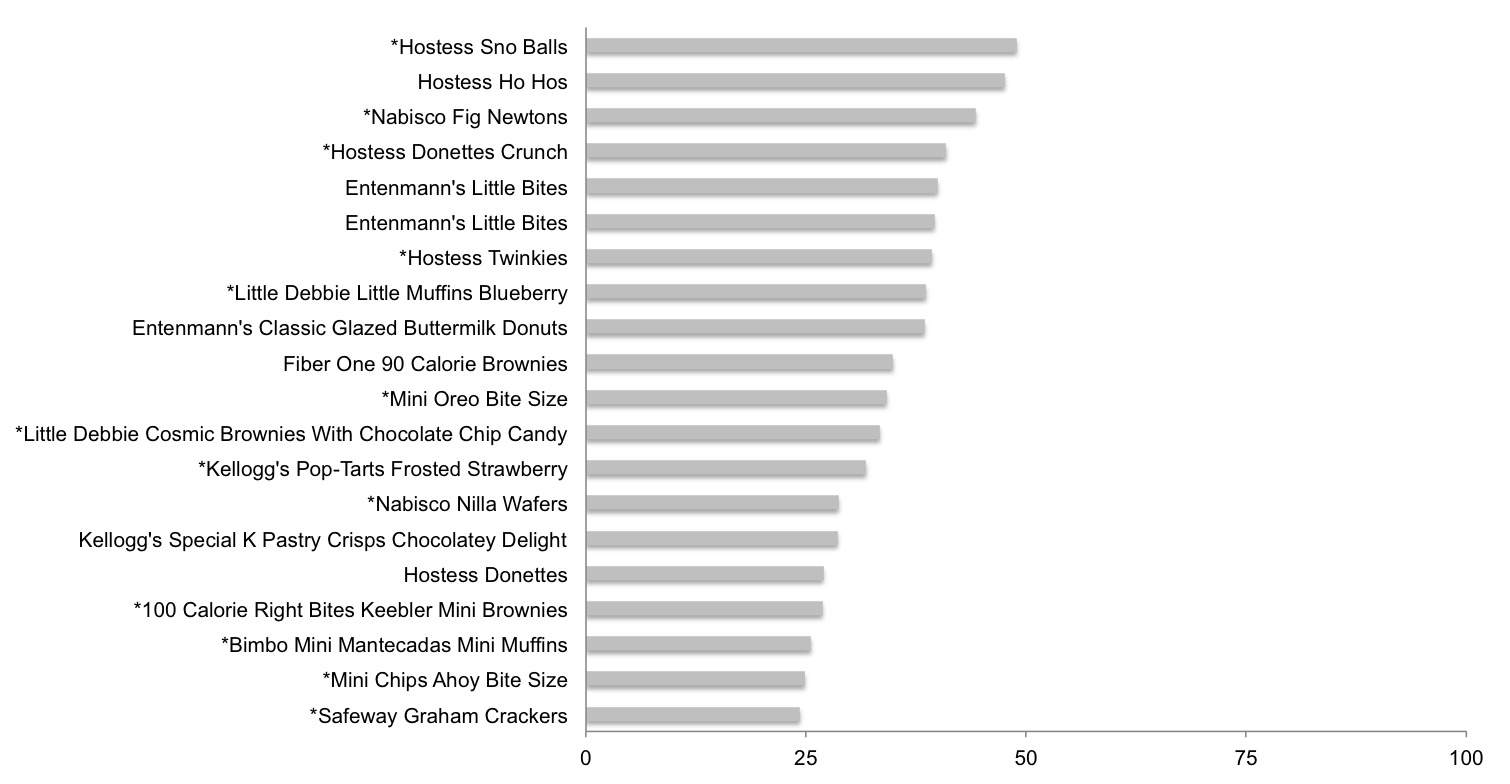


**Figure S2.** Percent of total calories from sugar: Packaged Baked Goods. ***** HFCS listed as ingredient.

**Table S1.** Formulas.

| Definitions: F = fructose, G = glucose, GAL = galactose, M = maltose, L = lactose, S = sucrose |
| --- |
| 1. Total Sugar_Actual_ per 100 g sample = S + L + M + G + F + GAL |
| 2. Total % sugar in sample = (*X* g sugar/Total Sugar_Actual_ per 100 g sample) × 100 |
| 3. Total Sugar_Actual_ per serving size = Total Sugar_Actual_ per 100 g/(100/*X* g/s) |
| 4. Amount individual sugars per serving = *X* g per 100 g/(100/*X* g/s) |
| 5. Grams G from disaccharide = grams M + (0.5 * grams L) |
| 6. F:G (raw) = [F grams/(F grams + G grams)] × 100 |
| 7. F:G (adjusted) = [F grams/(F grams + G grams +grams G from disaccharide )] × 100 |
| 8. % Total Calories from sugar = [(Total Sugar_Actual_ per serving × 4)/Total Calories per serving] × 100 |

* Sugar Calculations (based upon lab results provided in g/100 g sample format).

**Table S2.** Sources of Sugars.

| **Food Category** | **Sugar Listed on Label** | | | | | | | | | **Total Sugar Sources** | | | |
| --- | --- | --- | --- | --- | --- | --- | --- | --- | --- | --- | --- | --- | --- |
| **Baby Foods** | **None** | **HFCS** | **SUC** | **FRU** | **LAC** | **GLU** | **GAL** | **MAL** | **CS** | **% AS *** | **AS/100 g** | **% IS **** | **IS/100 g** |
| Gerber Whole Wheat Cereal For Baby | ✓ |  |  |  |  |  |  |  |  | 0 | 0 | 100 | 22.80 |
| Gerber Oatmeal Single Grain | ✓ |  |  |  |  |  |  |  |  | 0 | 0 | 100 | 4.50 |
| Gerber Rice Singe Grain | ✓ |  |  |  |  |  |  |  |  | 0 | 0 | 100 | 6.90 |
| Earth’s Best Organic Whole Grain Oatmeal Cereal | ✓ |  |  |  |  |  |  |  |  | 0 | 0 | 100 | 16.00 |
| Gerber Second Foods Nature Select Prunes With Apples | ✓ |  |  |  |  |  |  |  |  | 0 | 0 | 100 | 12.00 |
| Gerber Second Foods Spoonable Smoothies Hawaiian Delight | ✓ |  |  |  |  |  |  |  |  | 0 | 0 | 100 | 21.00 |
| First Foods Gerber Nature Select Apples | ✓ |  |  |  |  |  |  |  |  | 0 | 0 | 100 | 10.80 |
| Second Foods Gerber Spoonable Smoothies Peach Cobbler | ✓ |  |  |  |  |  |  |  |  | 0 | 0 | 100 | 12.20 |
| Earth’s Best Second Carrots | ✓ |  |  |  |  |  |  |  |  | 0 | 0 | 100 | 4.50 |
| O Organics (1) Organic Carrots | ✓ |  |  |  |  |  |  |  |  | 0 | 0 | 100 | 3.70 |
| Beech Nut Stage 2 Tender Sweet Carrots | ✓ |  |  |  |  |  |  |  |  | 0 | 0 | 100 | 3.80 |
| Gerber Oatmeal Single Grain Cereal For Baby | ✓ |  |  |  |  |  |  |  |  | 0 | 0 | 100 | 1.60 |
| Gerber Graduates Puffs Cereal Snack Peach |  |  | ✓ |  |  |  |  |  |  | 86.31 | 14.5 | 13.69 | 2.3 |
| Gerber Graduates Breakfast Buddies Apple Cinnamon |  |  | ✓ |  |  |  |  |  |  | 26.51 | 2.2 | 73.49 | 6.1 |
| Gerber Graduates For Preschoolers Juice Treats Fruit Medley |  |  | ✓ |  |  |  |  |  | ✓ | 66.67 | 40.6 | 33.33 | 20.3 |
| Gerber Yogurt Blends Peach |  |  | ✓ |  |  |  |  |  |  | 53.77 | 5.7 | 46.23 | 4.9 |
| **♯** Gerber Graduates For Toddlers Cereal Bars Strawberry Banana |  | ✓ | ✓ |  |  |  |  |  | ✓ |  |  |  |  |
| ♯ Gerber Graduates For Preschoolers Cereal Twists Banana Peach |  |  | ✓ | ✓ |  |  |  |  | ✓ |  |  |  |  |
| Gerber Graduates Grabbers Fruit & Yogurt |  |  | ✓ |  |  |  |  |  |  | 34.78 | 4 | 65.22 | 7.5 |
| **Baked Goods** | **None** | **HFCS** | **SUC** | **FRU** | **LAC** | **GLU** | **GAL** | **MAL** | **CS** | **% AS *** | **AS/100 g** | **% IS **** | **IS/100 g** |
| Kellogg’s Special K Pastry Crisps Chocolatey Delight |  |  | ✓ | ✓ |  |  |  |  |  | 79.37 | 22.7 | 20.63 | 5.9 |
| Entenmann’s Little Bites |  |  | ✓ | ✓ |  |  |  |  | ✓ | 92.75 | 37.1 | 7.25 | 2.9 |
| Hostess Ho Hos |  |  | ✓ |  |  |  |  |  | ✓ | 90.87 | 45.8 | 9.13 | 4.6 |
| Entenmann’s Classic Glazed Buttermilk Donuts |  |  | ✓ |  | ✓ |  |  |  |  | 95.80 | 38.8 | 4.20 | 1.7 |
| Hostess Donettes |  |  | ✓ |  |  |  |  |  |  | 52.94 | 14.4 | 47.06 | 12.8 |
| Entenmann’s Little Bites |  |  | ✓ | ✓ |  |  |  |  | ✓ | 94.97 | 41.5 | 5.03 | 2.2 |
| Fiber One 90 Calorie Brownies |  |  | ✓ | ✓ |  |  |  |  |  | 97.77 | 30.7 | 2.23 | 0.7 |

**Table S2.** *Cont.*

| **Food Category** | **Sugar Listed on Label** | **Total Sugar Sources** |
| --- | --- | --- |

| **Baked Goods *cont.*** | **None** | **HFCS** | **SUC** | **FRU** | **LAC** | **GLU** | **GAL** | **MAL** | **CS** | **% AS *** | **AS/100 g** | **% IS **** | **IS/100 g** |
| --- | --- | --- | --- | --- | --- | --- | --- | --- | --- | --- | --- | --- | --- |

| ♯ Little Debbie Little Muffins Blueberry |  | ✓ | ✓ |  |  |  |  |  |  |  |  |  |  |
| --- | --- | --- | --- | --- | --- | --- | --- | --- | --- | --- | --- | --- | --- |
| Nabisco Nilla Wafers |  | ✓ | ✓ |  |  |  |  |  |  | 96.42 | 32.3 | 3.58 | 1.2 |
| Safeway Graham Crackers |  | ✓ | ✓ |  |  |  |  |  |  | 98.82 | 25.2 | 1.18 | 0.3 |
| 100 Calorie Right Bites Keebler Mini Brownies |  | ✓ | ✓ | ✓ |  |  |  |  | ✓ | 98.44 | 31.5 | 1.56 | 0.5 |
| ♯ Kellogg’s Pop-Tarts Frosted Strawberry |  | ✓ | ✓ |  |  |  |  |  | ✓ |  |  |  |  |
| Hostess Donettes Crunch |  | ✓ | ✓ |  |  |  |  |  |  | 94.79 | 30.9 | 5.21 | 1.7 |
| Hostess Sno Balls |  | ✓ | ✓ |  |  |  |  |  | ✓ | 99.33 | 44.2 | 0.67 | 0.3 |
| Hostess Twinkies |  | ✓ | ✓ |  |  | ✓ |  |  | ✓ | 97.65 | 37.4 | 2.35 | 0.9 |
| Bimbo Mini Mantecadas Mini Muffins |  | ✓ | ✓ |  |  |  |  |  |  | 97.81 | 26.8 | 2.19 | 0.6 |
| Mini Chips Ahoy Bite Size |  | ✓ | ✓ |  |  |  |  |  |  | 100.00 | 31.1 | 0.00 | 0 |
| Mini Oreo Bite Size |  | ✓ | ✓ |  |  |  |  |  |  | 100.00 | 38.3 | 0.00 | 0 |
| ♯ Nabisco Fig Newtons |  | ✓ | ✓ |  |  |  |  |  | ✓ |  |  |  |  |
| Little Debbie Cosmic Brownies With Chocolate Chip Candy |  | ✓ | ✓ |  |  |  |  |  | ✓ | 100.00 | 37.7 | 0.00 | 0 |
| **Cereal** | **None** | **HFCS** | **SUC** | **FRU** | **LAC** | **GLU** | **GAL** | **MAL** | **CS** | **% AS *** | **AS/100 g** | **% IS **** | **IS/100 g** |
| Kashi Go Lean Crunch | ✓ |  |  |  |  |  |  |  |  | 0 | 0 | 100 | 22 |
| Lucky Charms |  |  | ✓ |  |  |  |  |  | ✓ | 84.40 | 36.8 | 15.60 | 6.8 |
| Cinnamon Toast Crunch |  |  | ✓ | ✓ |  |  |  |  |  | 94.50 | 29.2 | 5.50 | 1.7 |
| Frosted Cheerios |  |  | ✓ |  |  |  |  |  | ✓ | 94.20 | 32.5 | 5.80 | 2 |
| Golden Grahams |  |  | ✓ |  |  |  |  |  |  | 91.74 | 32.2 | 8.26 | 2.9 |
| Cheerios |  |  | ✓ |  |  |  |  |  |  | 97.50 | 3.9 | 2.50 | 0.1 |
| Honey Nut Cheerios |  |  | ✓ |  |  |  |  |  |  | 87.86 | 30.4 | 12.14 | 4.2 |
| Cocoa Puffs |  |  | ✓ | ✓ |  |  |  |  | ✓ | 93.91 | 37 | 6.09 | 2.4 |
| Trix |  |  | ✓ |  |  |  |  |  | ✓ | 86.96 | 28 | 13.04 | 4.2 |
| Honey Bunches Of Oats |  |  | ✓ |  |  |  |  |  | ✓ | 90.00 | 17.1 | 10.00 | 1.9 |
| Life |  |  | ✓ |  |  |  |  |  |  | 93.90 | 20 | 6.10 | 1.3 |
| Rice Chex |  |  | ✓ |  |  |  |  |  |  | 94.59 | 7 | 5.41 | 0.4 |
| Corn Flakes |  |  | ✓ |  |  |  |  |  |  | 59.52 | 5 | 40.48 | 3.4 |

**Table S2.** *Cont.*

| **Food Category** | **Sugar Listed on Label** | | | | | | | | | **Total Sugar Sources** | | | |
| --- | --- | --- | --- | --- | --- | --- | --- | --- | --- | --- | --- | --- | --- |
| **Cereal *cont.*** | **None** | **HFCS** | **SUC** | **FRU** | **LAC** | **GLU** | **GAL** | **MAL** | **CS** | **% AS *** | **AS/100 g** | **% IS **** | **IS/100 g** |

| Frosted Mini Wheats Little Bites |  |  | ✓ |  |  |  |  |  |  | 95.00 | 19 | 5.00 | 1 |
| --- | --- | --- | --- | --- | --- | --- | --- | --- | --- | --- | --- | --- | --- |

| Kellogg’s Special K |  |  | ✓ |  |  |  |  |  |  | 86.78 | 10.5 | 13.22 | 1.6 |
| --- | --- | --- | --- | --- | --- | --- | --- | --- | --- | --- | --- | --- | --- |
| Safeway Kitchens Crispy Rice |  |  | ✓ |  |  |  |  |  |  | 89.52 | 9.4 | 10.48 | 1.1 |
| Essential Everyday Crispy Rice |  | ✓ | ✓ |  |  |  |  |  |  | 96.47 | 8.2 | 3.53 | 0.3 |
| Kix |  |  | ✓ |  |  |  |  |  |  | 93.07 | 9.4 | 6.93 | 0.7 |
| Kellogg’s Froot Loops |  |  | ✓ |  |  |  |  |  |  | 97.80 | 40 | 2.20 | 0.9 |
| Kellogg’s Frosted Flakes |  |  | ✓ |  |  |  |  |  |  | 91.94 | 36.5 | 8.06 | 3.2 |
| **Formulas** | **None** | **HFCS** | **SUC** | **FRU** | **LAC** | **GLU** | **GAL** | **MAL** | **CS** | **% AS *** | **AS/100 g** | **% IS **** | **IS/100 g** |
| Similac Advance Complete Nutrition |  |  |  |  | ✓ |  |  |  |  | 98.41 | 6.2 | 1.59 | 6.30 |
| Similac Go & Grow Compete Toddler Nutrition, Infant Formula With Iron | ✓ |  |  |  |  |  |  |  |  | 0.00 | 0 | 100 | 48.10 |
| Gerber Good Start Soothe | ✓ |  |  |  |  |  |  |  |  | 0.00 | 0 | 100 | 5.90 |
| Parent’s Choice Infant Formula With Iron Premium Milk-Based |  |  |  |  | ✓ |  |  |  |  | 98.05 | 50.3 | 1.95 | 1.00 |
| Gerber Good Start Protect |  |  |  |  | ✓ |  |  |  |  | 96.62 | 37.2 | 3.38 | 1.30 |
| Gerber Good Start Gentle |  |  |  |  | ✓ |  |  |  |  | 92.90 | 32.7 | 7.10 | 2.50 |
| Enfamil Premium Infant |  |  |  |  | ✓ |  |  |  |  | 98.38 | 48.5 | 1.62 | 0.80 |
| Pediasure Shake Vanilla |  |  | ✓ |  |  |  |  |  |  | 98.63 | 7.2 | 1.37 | 0.1 |
| Similac Alimentum |  |  | ✓ |  |  |  |  |  |  | 100.00 | 4.4 | 0.00 | 0 |
| Enfagrow Premium Older Toddler |  |  | ✓ |  |  |  |  |  |  | 14.81 | 0.8 | 85.19 | 4.6 |
| Earth’s Best Organic Infant Formula With Iron |  |  |  |  | ✓ | ✓ |  |  | ✓ | 100.00 | 46.5 | 0.00 | 0 |
| Nutramigen With Enflora Lgg |  |  |  |  |  |  |  |  | ✓ | 57.78 | 2.6 | 42.22 | 1.9 |
| Enfagrow Gentlease Toddler |  |  |  |  |  |  |  |  | ✓ | 11.06 | 2.2 | 88.94 | 17.7 |
| Mom To Mom Sensitivity Infant Formula With Iron |  |  | ✓ |  |  |  |  |  | ✓ | 88.33 | 15.9 | 11.67 | 2.1 |
| Similac Advance Organic |  |  | ✓ |  |  |  |  |  |  | 44.68 | 14.7 | 55.32 | 18.2 |
| Parent’s Choice Infant Formula With Iron Soy-Based |  |  |  |  |  |  |  |  | ✓ | 4.60 | 0.8 | 95.40 | 16.6 |
| Parent’s Choice Infant Formula Milk Based With Iron Gentle |  |  |  |  |  |  |  |  | ✓ | 0.00 | 0 | 100.00 | 50.9 |
| Gerber Good Start Soy |  |  | ✓ |  |  |  |  |  |  | 82.14 | 11.5 | 17.86 | 2.5 |
| Similac Soy Isomil For Fussiness & Gas |  |  | ✓ |  |  |  |  |  | ✓ | 84.76 | 13.9 | 15.24 | 2.5 |

**Table S2.** *Cont.*

| **Food Category** | **Sugar Listed on Label** | | | | | | | | | **Total Sugar Sources** | | | |
| --- | --- | --- | --- | --- | --- | --- | --- | --- | --- | --- | --- | --- | --- |
| **Formulas *cont.*** | **None** | **HFCS** | **SUC** | **FRU** | **LAC** | **GLU** | **GAL** | **MAL** | **CS** | **% AS *** | **AS/100 g** | **% IS **** | **IS/100 g** |
| Similac Sensitive For Fussiness & Gas Due To Lactose Sensitivity |  |  | ✓ |  |  |  |  |  | ✓ | 87.97 | 25.6 | 12.03 | 3.5 |
| **Yogurt** | **None** | **HFCS** | **SUC** | **FRU** | **LAC** | **GLU** | **GAL** | **MAL** | **CS** | **% AS *** | **AS/100 g** | **% IS **** | **IS/100 g** |
| Chobani Raspberry | ✓ |  |  |  |  |  |  |  |  | 0 | 0 | 100 | 11.70 |
| Yoplait Gogurt (Perry Berry) |  |  | ✓ |  |  |  |  |  |  | 79.53 | 13.6 | 20.47 | 3.5 |
| Yoplait Gogurt (Summer Punch) |  |  | ✓ |  |  |  |  |  |  | 79.77 | 13.8 | 20.23 | 3.5 |
| ♯ Lucerne Lowfat Yogurt Tubes (Blueberry Bubblegum) |  |  | ✓ | ✓ |  |  |  |  |  |  |  |  |  |
| ♯ Lucerne Lowfat Yogurt Tubes (Strawberry) |  |  | ✓ | ✓ |  |  |  |  |  |  |  |  |  |
| Yoplait Trix Strawberry Banana Bash |  |  | ✓ | ✓ |  |  |  |  |  | 72.79 | 9.9 | 27.21 | 3.7 |
| Activia Vanilla |  |  | ✓ | ✓ |  |  |  |  |  | 72.73 | 10.4 | 27.27 | 3.9 |
| Activia Light Strawberry |  |  |  | ✓ |  |  |  |  |  |  |  |  |  |
| Yoplait Whips! Chocolate |  | ✓ | ✓ | ✓ |  |  |  |  |  | 78.28 | 15.5 | 21.72 | 4.3 |
| Yoplait Light Key Lime Pie |  |  | ✓ |  |  |  |  |  |  | 39.29 | 2.2 | 60.71 | 3.4 |
| Yoplait Light Banana Cream Pie |  |  | ✓ |  |  |  |  |  |  | 40.00 | 2.6 | 60.00 | 3.9 |
| Yoplait Original Strawberry |  |  | ✓ |  |  |  |  |  |  | 72.50 | 11.6 | 27.50 | 4.4 |
| Yoplait Light Very Vanilla |  |  | ✓ |  |  |  |  |  |  | 39.68 | 2.5 | 60.32 | 3.8 |
| Yoplait Light Blackberry |  |  | ✓ |  |  |  |  |  |  | 37.04 | 2 | 62.96 | 3.4 |
| Lucerne Low Fat Yogurt Peach |  |  | ✓ |  |  |  |  |  |  | 57.35 | 7.8 | 42.65 | 5.8 |
| Dannon Oikos Greek Nonfat Yogurt Vanilla |  |  | ✓ | ✓ |  |  |  |  |  | 76.92 | 9 | 23.08 | 2.7 |
| Dannon Danimal Smoothies Swingin’ Strawberry-Banana Flavored |  |  | ✓ |  |  |  |  |  |  | 76.64 | 10.5 | 23.36 | 3.2 |
| ♯ Great Value Strawberry Banana Nonfat Banana |  |  |  | ✓ |  |  |  |  |  |  |  |  |  |
| ♯ Dannon Oikos Blueberry |  |  | ✓ | ✓ |  |  |  |  |  |  |  |  |  |

Shaded rows indicate products with added sugar according to label. HFCS = high fructose corn syrup, SUC = sucrose, FRU = fructose, GAL = galactose, GLU = glucose, LAC = lactose, MAL = maltose, CS = corn syrup, AS = added sugar, IS = intrinsic sugar. ***** %TS from Added sugar = the sum of sugars listed on product label. ****** %TS from Intrinsic sugar = the sum of all other sugars. Items listing “sugar” as an ingredient include sucrose as an added sugar. Maltose is included as an added sugar in items containing corn syrup, as indicated on label. Glucose and fructose are included as added sugars in products listing HFCS on the label. ♯ In fruit-based products containing HFCS it is not possible to determine proportions of fructose and glucose from added *vs.* intrinsic sources.

**Table S3.** Sugar per Serving.

| **Infant Formula** **^♯,^^** | **SS** | **FRU (g/s)** | **GLU (g/s)** | **SUC (g/s)** | **MAL (g/s)** | **GAL (g/s)** | **LAC (g/s)** | **TS (g/s)** | **TS (g/100 g)** |
| --- | --- | --- | --- | --- | --- | --- | --- | --- | --- |
| Parent’s Choice Infant Formula With Iron Premium Milk-Based | 5FL OZ | 0.00 | 0.20 | 0.00 | 0.00 | 0.02 | 10.94 | 11.16 | 51.30 |
| Parent’s Choice Infant Formula Milk Based With Iron Gentle | 5 FL OZ | 0.00 | 0.20 | 0.00 | 0.00 | 0.02 | 10.85 | 11.07 | 50.9 |
| Enfamil Premium Infant | 5 FL OZ | 0.00 | 0.13 | 0.00 | 0.00 | 0.04 | 10.55 | 10.72 | 49.3 |
| Similac Go & Grow Compete Toddler Nutrition,  Infant Formula With Iron | 5 FL OZ | 0.00 | 0.22 | 0.00 | 0.00 | 0.02 | 10.22 | 10.46 | 48.1 |
| Earth’s Best Organic Infant Formula With Iron | 5 FL OZ | 0.00 | 0.07 | 0.00 | 0.35 | 0.00 | 9.70 | 10.11 | 46.5 |
| Gerber Good Start Protect | 5 FL OZ | 0.00 | 0.09 | 0.00 | 0.11 | 0.09 | 8.09 | 8.37 | 38.5 |
| Gerber Good Start Gentle | 5 FL OZ | 0.00 | 0.28 | 0.00 | 0.13 | 0.13 | 7.11 | 7.66 | 35.2 |
| Similac Advance Organic | 5 FL OZ | 0.04 | 0.11 | 3.20 | 0.24 | 0.00 | 3.57 | 7.16 | 32.9 |
| Similac Sensitive For Fussiness & Gas Due To Lactose Sensitivity | 5 FL OZ | 0.04 | 0.59 | 5.05 | 0.52 | 0.00 | 0.13 | 6.33 | 29.1 |
| Enfagrow Gentlease Toddler | 5FL OZ | 0.00 | 0.44 | 0.00 | 0.48 | 0.02 | 3.39 | 4.33 | 19.9 |
| Mom To Mom Sensitivity Infant Formula With Iron | 5 FL OZ | 0.02 | 0.26 | 2.50 | 0.96 | 0.00 | 0.17 | 3.92 | 18 |
| Parent’s Choice Infant Formula With Iron Soy-Based | 5 FL OZ | 0.00 | 0.07 | 0.00 | 0.17 | 0.11 | 3.44 | 3.78 | 17.4 |
| Similac Soy Isomil For Fussiness & Gas | 5 FL OZ | 0.04 | 0.50 | 2.31 | 0.72 | 0.00 | 0.00 | 3.57 | 16.4 |
| Gerber Good Start Soy | 5 FL OZ | 0.02 | 0.11 | 2.50 | 0.41 | 0.00 | 0.00 | 3.05 | 14 |
| Gerber Good Start Soothe | 5 FL OZ | 0.00 | 0.26 | 0.00 | 1.02 | 0.00 | 0.00 | 1.28 | 5.9 |
| * Pediasure Shake Vanilla | 8 FL OZ | - | - | - | - | - | - | - | 7.3 |
| * Similac Advance Complete Nutrition | 5 FL OZ | - | - | - | - | - | - | - | 6.3 |
| * Enfagrow Premium Older Toddler | 8.25 FL OZ | - | - | - | - | - | - | - | 5.4 |
| * Nutramigen With Enflora LGG | 5 FL OZ | - | - | - | - | - | - | - | 4.5 |
| * Similac Alimentum | 5 FL OZ | - | - | - | - | - | - | - | 4.4 |
| **Yogurt** | SS | FRU (g/s) | GLU (g/s) | SUC (g/s) | MAL (g/s) | GAL (g/s) | LAC (g/s) | TS (g/s) | TS (g/100 g) |
| Dannon Oikos Blueberry | 150 g | 8.85 | 1.80 | 4.65 | 0.00 | 0.90 | 3.00 | 19.20 | 12.80 |
| Dannon Oikos Greek N.F. Vanilla | 150 g | 7.05 | 0.45 | 6.45 | 0.00 | 0.90 | 2.70 | 17.55 | 11.70 |
| ^ **Yoplait Whips! Chocolate** | 113 g | 4.63 | 2.03 | 10.85 | 0.00 | 0.57 | 4.29 | 22.37 | 19.80 |
| Activia Vanilla | 113 g | 4.52 | 0.45 | 7.23 | 0.00 | 0.45 | 3.50 | 16.16 | 14.30 |
| Great Value N.F. Strawberry Banana | 170 g | 4.25 | 0.34 | 0.00 | 0.00 | 1.19 | 4.25 | 10.03 | 5.90 |
| ^ Lucerne L.F. Tubes Blueberry Bubblegum | 64 g | 2.62 | 0.13 | 4.48 | 0.00 | 0.26 | 2.50 | 9.98 | 15.60 |

**Table S3.** *Cont.*

| **Yogurt *cont.*** | **SS** | **FRU (g/s)** | **GLU (g/s)** | **SUC (g/s)** | **MAL (g/s)** | **GAL (g/s)** | **LAC (g/s)** | **TS (g/s)** | **TS (g/100 g)** |
| --- | --- | --- | --- | --- | --- | --- | --- | --- | --- |
| ^ Lucerne L.F. Tubes Strawberry | 64 g | 2.56 | 0.06 | 4.54 | 0.00 | 0.26 | 2.50 | 9.92 | 15.50 |
| Lucerne L.F. Peach | 170 g | 2.55 | 2.21 | 13.26 | 0.00 | 0.85 | 4.25 | 23.12 | 13.60 |
| Activia Light Strawberry | 113 g | 2.49 | 0.34 | 0.23 | 0.00 | 0.45 | 4.52 | 8.02 | 7.10 |
| Chobani Raspberry | 170 g | 2.04 | 1.70 | 11.73 | 0.00 | 0.85 | 3.57 | 19.89 | 11.70 |
| Yoplait Original Strawberry | 170 g | 0.68 | 0.68 | 19.72 | 0.00 | 0.85 | 5.27 | 27.20 | 16.00 |
| Yoplait Light Blackberry | 170 g | 0.34 | 0.34 | 3.40 | 0.00 | 0.68 | 4.42 | 9.18 | 5.40 |
| ^ Dannon Danimal Strawberry-Banana Flavored | 3.1 FL OZ | 0.29 | 0.29 | 10.15 | 0.00 | 0.48 | 2.03 | 13.25 | 13.70 |
| ^ Yoplait Gogurt Perry Berry | 64 g | 0.13 | 0.13 | 8.70 | 0.00 | 0.32 | 1.66 | 10.94 | 17.10 |
| ^ Yoplait Trix Strawberry Banana Bash | 113 g | 0.11 | 0.23 | 11.19 | 0.00 | 0.45 | 3.39 | 15.37 | 13.60 |
| ^ Yoplait Gogurt Summer Punch | 64 g | 0.06 | 0.13 | 8.83 | 0.00 | 0.32 | 1.73 | 11.07 | 17.30 |
| Yoplait Light Key Lime Pie | 170 g | 0.00 | 0.00 | 3.74 | 0.00 | 0.68 | 5.10 | 9.52 | 5.60 |
| Yoplait Light Banana Cream Pie | 170 g | 0.00 | 0.17 | 4.42 | 0.00 | 0.85 | 5.61 | 11.05 | 6.50 |
| Yoplait Light Very Vanilla | 170 g | 0.00 | 0.17 | 4.25 | 0.00 | 0.85 | 5.44 | 10.71 | 6.30 |
| **Packaged Baked Goods** | SS | FRU (g/s) | GLU (g/s) | SUC (g/s) | MAL (g/s) | GAL (g/s) | LAC (g/s) | TS (g/s) | TS (g/100 g) |
| **Nabisco Fig Newtons** | 31 g | 3.53 | 4.34 | 3.26 | 0.99 | 0.06 | 0.00 | 12.18 | 39.30 |
| ^ **Hostess Twinkies** | 77 g | 3.31 | 9.93 | 12.63 | 2.93 | 0.00 | 0.69 | 29.49 | 38.30 |
| **Keebler 100 Calorie Right Bites** | 21 g | 3.15 | 1.20 | 1.87 | 0.40 | 0.00 | 0.11 | 6.72 | 32.00 |
| Kellogg’s Special K Pastry Crisps | 25 g | 2.40 | 0.93 | 3.28 | 0.48 | 0.03 | 0.05 | 7.15 | 28.60 |
| ^ **Kellogg’s Pop-Tarts Strawberry** | 52 g | 2.29 | 8.37 | 3.64 | 1.61 | 0.00 | 0.00 | 15.91 | 30.60 |
| Fiber One 90 Calorie Brownies | 25 g | 1.78 | 0.15 | 5.90 | 0.03 | 0.00 | 0.00 | 7.85 | 31.40 |
| ^ **Hostess Sno Balls** | 99 g | 1.58 | 8.22 | 28.91 | 5.05 | 0.00 | 0.30 | 44.06 | 44.50 |
| ^ Entenmann’s Little Bites | 48 g | 1.49 | 0.58 | 17.76 | 0.67 | 0.00 | 0.48 | 20.98 | 43.70 |
| **Little Debbie Muffins Blueberry** | 47 g | 1.46 | 3.01 | 12.46 | 0.19 | 0.00 | 0.28 | 17.39 | 37.00 |
| ^ Hostess Ho Hos | 85 g | 0.77 | 1.96 | 37.74 | 1.19 | 0.00 | 1.19 | 42.84 | 50.40 |
| **Safeway Graham Crackers** | 31 g | 0.65 | 0.62 | 6.54 | 0.00 | 0.09 | 0.00 | 7.91 | 40.00 |
| **Bimbo Mini Muffins** | 63 g | 0.63 | 0.76 | 15.50 | 0.06 | 0.00 | 0.32 | 17.26 | 25.50 |
| **Little Debbie Cosmic Brownies** | 62 g | 0.62 | 9.05 | 10.97 | 2.73 | 0.00 | 0.00 | 23.37 | 27.40 |
| **Nabisco Nilla Wafers** | 30 g | 0.45 | 0.51 | 8.73 | 0.00 | 0.00 | 0.36 | 10.05 | 37.70 |

**Table S3.** *Cont.*

| **Packaged Baked Goods *cont.*** | **SS** | **FRU (g/s)** | **GLU (g/s)** | **SUC (g/s)** | **MAL (g/s)** | **GAL (g/s)** | **LAC (g/s)** | **TS (g/s)** | **TS (g/100 g)** |
| --- | --- | --- | --- | --- | --- | --- | --- | --- | --- |
| ^ **Hostess Donettes Crunch** | 113 g | 0.34 | 1.47 | 33.11 | 1.47 | 0.00 | 0.45 | 36.84 | 33.50 |
| ^ **Mini Oreo Bite Size** | 29 g | 0.29 | 1.22 | 9.60 | 0.00 | 0.00 | 0.00 | 11.11 | 32.60 |
| Entenmann’s Buttermilk Donuts | 57 g | 0.23 | 0.57 | 21.77 | 0.17 | 0.00 | 0.34 | 23.09 | 38.30 |
| **Mini Chips Ahoy Bite Size** | 30 g | 0.18 | 0.39 | 8.76 | 0.00 | 0.00 | 0.00 | 9.33 | 40.50 |
| Hostess Donettes | 57 g | 0.11 | 6.50 | 8.21 | 0.40 | 0.00 | 0.29 | 15.50 | 31.10 |
| **Breakfast Cereal** | SS | FRU (g/s) | GLU (g/s) | SUC (g/s) | MAL (g/s) | GAL (g/s) | LAC (g/s) | TS (g/s) | TS (g/100 g) |
| ^ Cinnamon Toast Crunch | 39 g | 3.78 | 0.66 | 7.61 | 0.00 | 0.00 | 0.00 | 12.05 | 30.90 |
| ^ Kellogg’s Frosted Flakes | 60 g | 0.78 | 0.90 | 21.90 | 0.24 | 0.00 | 0.00 | 23.82 | 39.70 |
| ^ Honey Nut Cheerios | 32 g | 0.67 | 0.67 | 9.73 | 0.00 | 0.00 | 0.00 | 11.07 | 34.60 |
| Kashi Go Lean Crunch | 53 g | 0.42 | 0.74 | 8.90 | 1.59 | 0.00 | 0.00 | 11.66 | 22.00 |
| ^ Golden Grahams | 31 g | 0.40 | 0.43 | 9.98 | 0.00 | 0.06 | 0.00 | 10.88 | 35.10 |
| Corn Flakes | 28 g | 0.36 | 0.34 | 1.40 | 0.25 | 0.00 | 0.00 | 2.35 | 8.40 |
| ^ Cocoa Puffs | 27 g | 0.27 | 0.65 | 9.32 | 0.41 | 0.00 | 0.00 | 10.64 | 39.40 |
| ^ Trix | 31 g | 0.25 | 1.05 | 8.12 | 0.56 | 0.00 | 0.00 | 9.98 | 32.20 |
| ^ Lucky Charms | 27 g | 0.24 | 1.59 | 9.37 | 0.57 | 0.00 | 0.00 | 11.77 | 43.60 |
| **Essential Everyday Crispy Rice** | 33 g | 0.23 | 0.26 | 2.21 | 0.10 | 0.00 | 0.00 | 2.81 | 8.50 |
| Honey Bunches Of Oats | 30 g | 0.21 | 0.30 | 4.65 | 0.48 | 0.00 | 0.06 | 5.70 | 19.00 |
| ^ Kellogg’s Froot Loops | 42 g | 0.17 | 0.21 | 16.80 | 0.00 | 0.00 | 0.00 | 17.18 | 40.90 |
| Kellogg’s Special K | 31 g | 0.16 | 0.12 | 3.26 | 0.19 | 0.00 | 0.03 | 3.75 | 12.10 |
| ^ Kix | 30 g | 0.12 | 0.09 | 2.82 | 0.00 | 0.00 | 0.00 | 3.03 | 10.10 |
| ^ Frosted Cheerios | 28 g | 0.11 | 0.45 | 8.88 | 0.22 | 0.00 | 0.00 | 9.66 | 34.50 |
| Frosted Mini Wheats | 55 g | 0.11 | 0.17 | 10.45 | 0.28 | 0.00 | 0.00 | 11.00 | 20.00 |
| Safeway Crispy Rice | 33 g | 0.10 | 0.13 | 3.10 | 0.13 | 0.00 | 0.00 | 3.47 | 10.50 |
| ^ Life | 32 g | 0.06 | 0.10 | 6.40 | 0.26 | 0.00 | 0.00 | 6.82 | 21.30 |
| Rice Chex | 27 g | 0.05 | 0.05 | 1.89 | 0.00 | 0.00 | 0.00 | 2.00 | 7.40 |
| ^ Cheerios | 28 g | 0.03 | 0.00 | 1.09 | 0.00 | 0.00 | 0.00 | 1.12 | 4.00 |
| **Baby Food ^** | SS | FRU (g/s) | GLU (g/s) | SUC (g/s) | MAL (g/s) | GAL (g/s) | LAC (g/s) | TS (g/s) | TS (g/100 g) |
| Gerber Smoothies Hawaiian Delight | 99 g | 8.12 | 7.33 | 3.76 | 0.00 | 0.10 | 1.49 | 20.79 | 21.00 |

**Table S3.** *Cont.*

| **Baby Food ^ *cont.*** | **SS** | **FRU (g/s)** | **GLU (g/s)** | **SUC (g/s)** | **MAL (g/s)** | **GAL (g/s)** | **LAC (g/s)** | **TS (g/s)** | **TS (g/100 g)** |
| --- | --- | --- | --- | --- | --- | --- | --- | --- | --- |
| Gerber Prunes With Apples | 99 g | 6.44 | 5.05 | 0.40 | 0.00 | 0.00 | 0.00 | 11.88 | 12.00 |
| Gerber Smoothies Peach Cobbler | 99 g | 5.64 | 4.85 | 1.58 | 0.00 | 0.00 | 0.00 | 12.08 | 12.20 |
| Gerber Nature Select Apples | 71 g | 5.25 | 1.78 | 0.64 | 0.00 | 0.00 | 0.00 | 7.67 | 10.80 |
| Gerber Grabbers Fruit & Yogurt | 120 g | 4.80 | 2.52 | 4.80 | 0.00 | 0.36 | 1.32 | 13.80 | 11.50 |
| Gerber Breakfast Apple Cinnamon | 128 g | 4.48 | 3.33 | 2.82 | 0.00 | 0.00 | 0.00 | 10.62 | 8.30 |
| Gerber Juice Treats Fruit Medley | 28 g | 2.16 | 3.53 | 10.22 | 1.15 | 0.00 | 0.00 | 17.05 | 60.90 |
| **Gerber Cereal Bars Strawberry Banana** | 19 g | 1.67 | 2.34 | 3.06 | 0.17 | 0.00 | 0.04 | 7.28 | 38.30 |
| Earth’s Best Second Carrots | 113 g | 1.24 | 1.24 | 2.60 | 0.00 | 0.00 | 0.00 | 5.09 | 4.50 |
| Gerber Cereal Twists Banana Peach | 20 g | 0.66 | 1.20 | 3.84 | 0.34 | 0.00 | 0.16 | 6.20 | 31.00 |
| Beech Nut Tender Sweet Carrots | 113 g | 0.57 | 0.57 | 3.16 | 0.00 | 0.00 | 0.00 | 4.29 | 3.80 |
| Gerber Yogurt Blends Peach | 99 g | 0.50 | 0.40 | 5.64 | 0.00 | 0.69 | 3.27 | 10.49 | 10.60 |
| O Organics (1) Organic Carrots | 71 g | 0.36 | 0.43 | 1.85 | 0.00 | 0.00 | 0.00 | 2.63 | 3.70 |
| Gerber Puffs Cereal Snack Peach | 7 g | 0.12 | 0.04 | 1.02 | 0.00 | 0.00 | 0.00 | 1.18 | 16.80 |
| Gerber Whole Wheat Cereal (Baby) | 16 g | 0.02 | 0.05 | 0.18 | 3.41 | 0.00 | 0.00 | 3.65 | 22.80 |
| Gerber Oatmeal Single Grain | 15 g | 0.00 | 0.00 | 0.15 | 0.53 | 0.00 | 0.00 | 0.68 | 0.60 |
| Gerber Rice Singe Grain | 16 g | 0.00 | 0.46 | 0.03 | 0.61 | 0.00 | 0.00 | 1.10 | 4.50 |
| Earth’s Best Organic Whole Grain Oatmeal | 14 g | 0.00 | 0.03 | 0.13 | 2.09 | 0.00 | 0.00 | 2.24 | 6.90 |
| Gerber Oatmeal Single Grain Cereal (Baby) | 15 g | 0.00 | 0.00 | 0.14 | 0.11 | 0.00 | 0.00 | 0.24 | 16.00 |

Sugar concentrations by per serving size and per liter values. Bold print represents products listing HFCS as an ingredient. ^ = products directly marketed towards children based upon packaging and advertising. SS = serving size, FRU = fructose, GAL = galactose, GLU = glucose, LAC = lactose, MAL = maltose, SUC = sucrose, TS = total sugar, g/s = grams per serving. To calculate Total Fructose (including from sucrose) use the following equation: Fructose_total_ = [0.5 × Sucrose g/s] + Fructose g/s; **♯** Formula sugar-per-serving values based on mixing instructions on label for a 5 fl oz serving size. Gas chromatography sugar values were provided on a g of sugar/100 g of sample basis. Values were normalized to reflect dry weight of mixed formula (Example: 5 FL OZ serving size = 2.5, 8.7 g scoops of powdered formula or 21.75 g of formula).
* Unable to calculate grams per serving in premixed, ready to use samples.

© 2015 by the authors; licensee MDPI, Basel, Switzerland. This article is an open access article distributed under the terms and conditions of the Creative Commons Attribution license (http://creativecommons.org/licenses/by/4.0/).
